# The Evolution of the Modern Hospital

**Published:** 1915-03-27

**Authors:** B. Burford Rawlings


### THE EVOLUTION OF THE MODERN HOSPITAL.*

### IV.—Medical Education, Administration, and Finance.

By B. BURFORD RAWLINGS.

To assume, as medical writers often do, that inquiry by laymen into medical methods in hospitals is impertinent, and indicates want of appreciation, appears an inadequate way of meeting criticism. Every public worker must be prepared for critical observation, and the language of denunciation is usually applied by opponents in the exact ratio of his distinction. None but the insignificant is immune. A leading morning paper charges the Prime Minister with "bamboozling the public," while another classifies a member of the Cabinet among "low organisms" who "possess a certain pernicious vitality like the microbes of some foul diseases." Both the Prime Minister and his colleague keep silence and survive. Why, then, a display of restiveness under notice by the spokesman of a profession which, though the practical application of its code of ethics may sometimes invite animadversion, commands an abundance of sincere eulogy? The staffs of the several hospitals do not make up the medical world. They are but a small, if distinguished, section of it, and their relationship to the whole body of medical workers is in itself a subject worthy of attention. But while that aspect of a big question affects chiefly the profession, the commanding position occupied by hospital consultants is a matter of moment to the community. It calls for careful investigation, and no effort to perfect hospital administration can command complete success unless it aims at setting the institutions free from the virtual proprietorship of a small body of men whose relation to the hospitals they serve is analogous to nothing in any other field of activity, and whose eminence is very largely due to the advantages of their hospital association.

### The Suggested Royal Commission.

More than thirty years ago, efforts were made to induce the Government to grant an inquiry by Royal Commission into the administration of the London hospitals, and at a conference lasting over several days, largely attended by men associated prominently with hospitals, cognate questions in great variety were debated. There was much insistence upon the need of facilitating the use of the hospitals for the purposes of the schools, and the value of the work's educational side. On the other hand, a layman representing a great general hospital with school attached, speaking as an active member of the governing body, gave expression to views commonly held by those behind the scenes when he said "that the relation between the curing of the sick and the education of students was a very important subject, and if a public inquiry ever took place into the medical

* Previous articles: February 20, March 6, 13 and 20.

schools; if people had their attention thoroughly drawn to the relations between the curing of disease and the education of medical men, it would be a lucky thing if there were not a great row." Nothing came of the conference. The Government refused the request for the Royal Commission which alone could deal satisfactorily with the complex issues involved, and while the prospect of the school being brought under the government of the hospital has been receding year by year, the disposition grows to make the hospital subservient to the school.

### The Lords' Inquiry.

The Select Committee of the House of Lords appointed some years later made the only inquiry into hospital administration within living memory. The attitude of the Committee well reflected the common sentiment. From the first, all serious reference to the medical work was avoided. Whole days were spent in listening to reiterated tales of the personal grievances of nurses; the towels in use were sedulously counted, and many ways of adding to expenditure were devised, but the dominating questions of finance and the relationship of staff and school to hospital were ignored. The verdict of the *Times* upon the Committee's report bore out this view. "The Committee was appointed to furnish the Government with good reasons for leaving the hospitals alone." No result beyond an all-round rise of expenditure followed upon the proceedings, and the relations of staff, school, and hospital have continued as before, but with a difference. Medical pretensions have advanced largely during the years which have passed since, and we may reasonably believe that the attitude of the Committee lent encouragement to professional ambitions. So, too, did the judgment of Sir Edward Fry's Committee after an inquiry held some years later into the affairs of the Queen Square Hospital. Efforts to tighten the medical hold upon hospitals and to exalt their scientific and educational character have been increased, and those who have at heart the welfare of hospitals as charitable agencies may well mark the progress of events with concern.

At a prize distribution to students of St. Bartholomew's, a late treasurer, then newly elected, boldly asserted that "the hospital and medical school are one, but if he were asked which was the more important of the two, he would say the school." This pronouncement was the more dangerous because it emanated from a layman, and its abstract truth is capable of hard mathematical demonstration. It went beyond anything openly advanced thitherto, and appears ill-calculated to stimulate subscriptions. Taken literally, it would carry a meaning which the speaker must certainly, and righteously, repudiate as slanderous of a beneficent institution whose well-won reputation gives it the lie.

### What Placing the School First Involves.

It would mean that the hospital, with all its vast machinery founded and maintained for the benefit of the patients treated within its walls, is first of all a scientific laboratory, where the bodies and sufferings of one set of human beings are exploited —under the guise of treatment—for the benefit of another set who are able to pay fees. It would mean that all the bright surroundings and tender ministrations of the hospital are but embellishments by which helpless patients are beguiled into a surrender of their bodies, and that the solicitude displayed for their comfort is the measure of an organised deception. It is not their well-being with which the hospital is chiefly concerned, but the advancement of science and the problematic mitigation of suffering other than their own. Fortunately for the credit of hospitals, and the safety of their patients, the attitude of the large majority of the men who compose the several staffs is scrupulous, humane, and sympathetic. But if lay authorities are to be found who put the school before the hospital, it may be believed that the mind of the master in science may sometimes overlook the alleviation of individual sufferings to fix his attention upon possibilities affecting the human race. Temptations intrude upon ardent physiologists as upon other pioneers. They prove fascinating to a degree undreamed of by those not subject to them, and lead not infrequently to the voluntary and deliberate sacrifice of the investigator's own body. If, in respect of hospital patients, resistance is commonplace, the fact testifies to the high character of the exponents of scientific medicine and surgery as a body, but it affords no protection from individual aberration.

